# Between-class variation and compositional correlates of physical fitness: a multilevel analysis of annual surveillance records from one Chinese university

**DOI:** 10.3389/fpsyg.2026.1892473

**Published:** 2026-08-18

**Authors:** Yan Bu, Junhui Zhu, Lin Wang, Mingling Zhao, YueQiu Li

**Affiliations:** 1 College of Physical Education and Health Science, Yibin University, Yibin, Sichuan, China; 2 Department of Marine Sports, Pukyong National University, Busan, Republic of Korea; 3 Faculty of Education, Universiti Kebangsaan Malaysia, Bangi, Selangor, Malaysia; 4 School of Urban Construction, Chengdu Polytechnic, Chengdu, Sichuan, China; 5 College of Sports and Great Health, Sichuan University of Business and Technology, Chengdu, Sichuan, China

**Keywords:** between-class variation, body mass index, class context, compositional effects, multilevel modeling, physical fitness, university students

## Abstract

This retrospective study quantified between-class variation in university students' physical fitness and examined individual- and class-level correlates, using annual surveillance records collected from 2020 to 2024 at one public university in Sichuan Province, China. Records were nested within class-year units (a specific administrative class observed in a specific survey year). Although no psychosocial constructs were measured directly, quantifying between-class variation is a necessary first step for health-psychology research on how peer norms, motivational climate, and collective efficacy may relate to student fitness; the study is therefore hypothesis-generating rather than a test of mechanisms. A total of 106,461 de-identified records from 2,591 class-year units were analyzed (44,240 male, 62,221 female; mean age 20.67 ± 1.54 years; mean BMI 21.64 ± 3.52 kg/m^2^). Multilevel linear models were fitted, with calendar year (2020–2024) entered as a categorical fixed-effect set in the primary specification. Between-class variation was substantial (null-model intraclass correlation coefficient [ICC] = 0.178; adjusted ICC after individual and calendar-year adjustment = 0.151). Higher within-class BMI, older age, and male sex were associated with lower fitness (within-class BMI β = −0.528, *p* < 0.001; sex [male vs. female] β = −4.174, *p* < 0.001); second- and fourth-year students scored higher than first-year students (β = 0.803 and 1.913). At the class level, a higher proportion of male students (β = 1.090, *p* < 0.001) was positively associated with fitness, whereas larger class size (β = −0.012, *p* = 0.021; a practically negligible 0.24-point difference across a 20-student gap) and higher mean class BMI (β = −0.906, *p* < 0.001, the largest class-level association) were associated with lower fitness. Standardized coefficients confirmed that sex and within-class BMI were the strongest individual-level correlates; the fixed effects explained 15.4% of the variance (marginal *R*^2^ = 0.154; conditional *R*^2^ = 0.282). A random-slope extension showed that the within-class BMI-fitness association varied across classes (likelihood-ratio χ^2^ = 463.07, df = 2, *p* < 0.001), but this heterogeneity was modest in practical terms (slope SD ≈ 0.27; ~95% of class-specific slopes between −1.05 and +0.01). Findings provide evidence of meaningful class-level variation—not a test of mechanisms—and indicate that analyses and interventions targeting university physical fitness should account for the clustered structure of the data.

## 1 Introduction

Physical fitness among university students is an important composite indicator of physical and psychological development in young adults and a key criterion for evaluating health promotion and talent cultivation in higher-education institutions. Nationally, student physical fitness in China continues to face substantial challenges, including fluctuations in endurance, strength, and speed indicators and the coexistence of overweight and obesity with insufficient fitness. Surveillance evidence based on five successive national surveys indicates that overall fitness among Chinese college students changed unfavorably alongside rising cardiovascular risk between 2000 and 2019, suggesting that the university period has become a critical window in which health risks emerge earlier in the life course (Cai et al., 2025). Because fitness is shaped not only by individual characteristics but also by the settings in which students study and exercise, understanding this problem requires attention to the organizational contexts—such as the class—in which students are embedded (Hu et al., 2021).

Substantial evidence exists on individual-level determinants of student fitness. The BMI-fitness association is consistently inverse and often non-linear: cross-sectional and multicentre studies of Chinese university students report that both underweight and, especially, overweight and obesity are associated with poorer performance on vital capacity, speed, endurance, and overall fitness, and that even normal-weight obesity is linked to lower fitness (Chen et al., 2020; Wu et al., 2024; Zhang et al., 2018). Sex and academic grade are likewise closely related to fitness levels. However, most studies of university-student fitness treat the individual student as the sole unit of analysis—relying on ordinary regression, between-group comparisons, or descriptive trends (e.g., Chen et al., 2020; Wu et al., 2024)—and pay limited attention to the hierarchical structure in which students are nested within classes, majors, and colleges. The comparatively few multilevel studies in Chinese educational settings (Cui et al., 2024; Zhou et al., 2025) illustrate the value of modeling this structure explicitly.

From an educational-organization perspective, the class is not merely a statistical “background variable” but a micro-institutional context in which students' daily learning, sport participation, peer interaction, and behavioral norms are formed and reproduced. This proximal setting is theoretically relevant to health psychology: the class environment may shape health behavior through peer norms and shared routines, through a collectively experienced motivational climate (Ryan and Deci, 2000), and through collective efficacy—a group's shared belief in its capacity to organize and execute action (Bandura, 1997; Zaccaro et al., 1995). Under the social-ecological model, individual behavior and health are jointly shaped by individual, interpersonal, organizational, and institutional levels (Hu et al., 2021), so that proximal social contexts such as the class can contribute meaningfully to individual outcomes. Consistent with this view, Novak et al. (2026) reported substantial school-level variation in youth fitness that remained after adjustment for individual covariates such as sex and class, and research on Chinese school physical-education contexts shows that class-level factors such as class size, lesson location, and lesson content influence the moderate-to-vigorous activity students accumulate (Zhou et al., 2025); cross-level associations among technology use, engagement, and fitness have likewise been observed in university physical education (Cui et al., 2024).

We emphasize at the outset that the class-level indicators available in the present surveillance database (class size, proportion of male students, mean class BMI) capture class composition rather than directly measured organizational or psychosocial context. The study is therefore exploratory and hypothesis-generating. Against this background, the study had three specific aims: (1) to quantify between-class variation in total physical-fitness scores (the ICC); (2) to examine whether class size, the proportion of male students, and mean class BMI are associated with fitness after adjustment for individual characteristics and calendar year; and (3) to test whether the within-class BMI-fitness slope varies across classes. Findings are intended to provide population-level evidence of class-level variation that can inform future studies—including those that directly measure class-level psychosocial and behavioral mechanisms—rather than to test such mechanisms here.

## 2 Materials and methods

### 2.1 Data source and study population

This was a retrospective observational analysis of annual physical-fitness surveillance records collected from 2020 to 2024 at one public university in Sichuan Province, Southwest China. The source database contained grade code, class code, class name, a within-year student identifier, sex, date of birth, height, weight, and, for each fitness component, the raw result, standardized score, and grade, together with bonus points and the total physical-fitness score. All testing followed the National Student Physical Fitness Standards (NSPFS). Under the NSPFS, the total score (0–120, with a bonus component; higher = better) is a weighted composite of body mass index (15%), vital capacity (15%), a 50-m sprint (20%), sit-and-reach flexibility (10%), standing long jump (10%), and an endurance/strength block (30%; the 800-m run and 1-min sit-ups for women, the 1000-m run and pull-ups for men), with sex- and, where applicable, grade-specific scoring norms. Because scoring norms are sex-specific, the total score is already partly adjusted for sex.

The source surveillance files contained administrative student identifiers. Before statistical modeling, direct identifiers were removed or transformed, and the analytic dataset retained only the variables required for analysis; stable cross-year identifiers were not available to the research team. The de-identified records were made available for research on 16 April, 2026. Because a stable cross-year identifier that could deterministically link the same student across the 2020–2024 window was not available, and because identifier-assignment rules differed across cohorts and academic years, longitudinal linkage of individual students across years was not possible. Obtaining stable personal identifiers would exceed the scope of the approved ethics protocol and the institutional data-protection agreement, and re-identification was therefore neither permitted nor feasible. Consequently, the annual record was adopted as the analytic unit, and within-student correlation across years could not be modeled directly; this limitation is addressed through sensitivity analyses (Section 2.4) and in the Discussion. The class-level unit was defined as a class-year unit—a specific administrative class observed in a specific survey year—because the student membership of a given administrative class changes across years as cohorts enter and leave.

**Figure 1:**
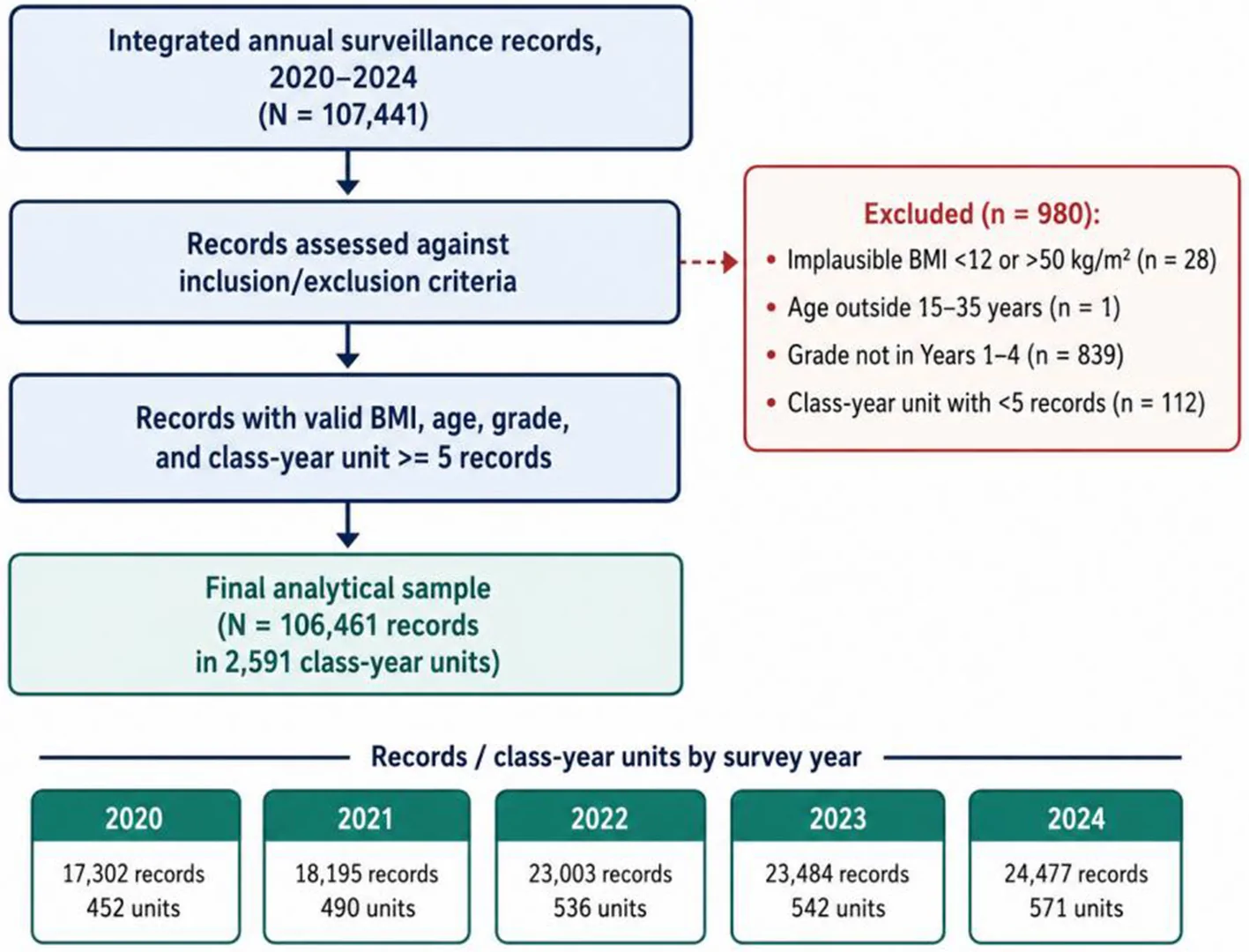
Flow of records through data integration and cleaning (107,441 → 106,461 records analyzed in 2,591 class-year units), with the by-year breakdown of the final sample.

After integration, 107,441 records were obtained. Records were excluded for implausible BMI (*n* = 28), abnormal age (*n* = 1), unidentifiable academic grade (*n* = 839), and membership in a class-year unit with fewer than five eligible records (*n* = 112), leaving 106,461 eligible records from 2,591 class-year units (Figure 1). The BMI bounds of 12–50 kg/m^2^ were used as physiological plausibility limits: values below 12 kg/m^2^ are incompatible with survival in ambulatory young adults, and values above 50 kg/m^2^ in this age band almost always reflect height/weight data-entry errors; the age window of 15–35 years excludes clerical date-of-birth errors while retaining the full plausible range for undergraduates. In the retained sample the median class-year size was 41 (range 5–84). No variables other than those listed had missing values relevant to the analysis (total scores were complete).

### 2.2 Variable definitions

#### 2.2.1 Dependent variable

The NSPFS total physical-fitness score, treated as continuous.

#### 2.2.2 Student-level variables

Sex, BMI, age, and academic grade were level-1 predictors. Age was computed in years from date of birth to 30 September of the survey year. Academic grade (Year 1–4) was derived from the entry year encoded in the student identifier and the survey year (grade = survey year – entry year + 1). BMI was computed as weight (kg) divided by height (m) squared.

#### 2.2.3 Class-level variables

*Three* level-2 variables were computed within each class-year unit: class size, proportion of male students, and mean class BMI. Calendar year (2020–2024) was entered as a record-level categorical fixed-effect set (dummy coded, 2020 reference).

### 2.3 Centring and interpretation

Individual BMI was centered around the mean BMI of its class-year unit (within-class centring), and the class mean BMI was entered as a separate level-2 predictor, so that within-class and between-class BMI associations could be separated—an approach recommended when contextual and individual associations may differ (Enders and Tofighi, 2007). The within-class BMI coefficient represents the association between a student's deviation from the class-year mean BMI and that student's fitness score; the class-mean BMI coefficient represents the association between a unit's average BMI and fitness scores after adjustment for individual deviation. Age, class size, proportion of male students, and mean class BMI were grand-mean centered so that the intercept refers to an average record in an average class-year unit; sex was coded male = 1, female = 0, and grade and calendar year were dummy coded.

### 2.4 Statistical analysis

Descriptive statistics were computed (mean ± SD for continuous variables; counts and proportions for categorical variables), and included vs. excluded records were compared. Analyses used Python 3.12 with statsmodels 0.14.6 (MixedLM); all models were fitted by maximum likelihood (ML) to permit direct AIC/BIC and likelihood-ratio comparison across nested models. A null random-intercept model estimated the between-class variance and the ICC:


$$\begin{array}{r} \text{Level }1:Y_{\text{ij}}=\beta_{0\text{j}}+r_{\text{ij}}\text{ Level }2:\beta_{0\text{j}}=\gamma_{00}+u_{0\text{j}}\\ \text{ICC}=\tau_{00}/\left(\tau_{00}+\;\sigma^{2}\right) \end{array}$$

where Y_ij_ is the total score for record i in unit j, r_ij_ the level-1 residual, u_0j_ the class-level random intercept, τ_00_ the between-class variance, and σ^2^ the residual variance.

Three nested specifications were compared: Model 1 (random intercept with individual- and class-level covariates, no calendar-year adjustment); Model 2 (Model 1 plus calendar-year fixed effects; the primary specification); and Model 3 (Model 2 with a random slope for within-class BMI). Calendar year was modeled categorically rather than as a linear trend because the 2020–2024 window spanned pandemic disruption (2020–2022), return-to-campus transitions, and possible changes in test administration and scoring (2023–2024); a linear term would impose a monotonic assumption inconsistent with these non-monotonic shifts. Model 2 was retained as the primary specification—rather than the better-fitting Model 3—because the random intercept-slope covariance in Model 3 was not statistically significant (Section 3.6), so the simpler random-intercept structure is preferred for inference on the fixed effects, with Model 3 reported as a robustness check documenting slope heterogeneity.

Fixed-effect coefficients, standard errors, 95% confidence intervals, standardized coefficients, variance components, AIC, BIC, log-likelihood, and marginal and conditional *R*^2^ (Nakagawa and Schielzeth, 2013) were reported. Nested-model comparisons used likelihood-ratio (LR) tests: Model 1 vs. null (Δdf = 9), Model 2 vs. Model 1 (Δdf = 4), and Model 3 vs. Model 2 (Δdf = 2). Model diagnostics comprised variance inflation factors (VIFs) for all fixed effects, residual-vs.-fitted and normal Q-Q plots, and a cluster-level influence analysis in which class-year units with standardized random intercepts beyond ±3 SD were flagged and the model refitted after their removal. To address the impossibility of cross-year linkage, a single-year sensitivity analysis was run within each survey year (each student contributing at most one record). Additional sensitivity models added mean class age as a level-2 predictor and, separately, removed age or grade to probe age-grade confounding. Supplementary material includes: (a) single-year sensitivity results for all five years; (b) model-diagnostic plots; (c) VIFs; and (d) random-intercept and random-slope distributions.

### 2.5 Ethics statement

This study was approved by the Institutional Review Board of the Science and Technology Ethics Committee of Yibin University (approval No. YBULLXS26041601). The requirement for written informed consent was waived by the Institutional Review Board because the analysis used de-identified routine surveillance records and posed no more than minimal risk to participants. All procedures were conducted in accordance with the Declaration of Helsinki and the relevant national and institutional requirements for the secondary analysis of de-identified administrative data. Yibin University was the approving body as the institution responsible for the data.

## 3 Results

### 3.1 Sample characteristics

The analytic sample comprised 106,461 records from 2,591 class-year units. Mean age was 20.67 ± 1.54 years and mean BMI 21.64 ± 3.52 kg/m^2^; 44,240 records (41.6%) were from male and 62,221 (58.4%) from female students. The mean total physical-fitness score was 71.60 ± 8.56. Male students had higher mean BMI (22.39 vs. 21.11 kg/m^2^) and lower total scores (68.80 vs. 73.59) than female students, consistent with the sex-specific NSPFS scoring norms. The median class-year size was 41 (mean 41.09 ± 12.63; range 5–84); the mean within-unit proportion of male students was 0.406 ± 0.257 and the mean class BMI 21.63 ± 0.81 kg/m^2^. Sample sizes were broadly comparable across survey years, ranging from 17,302 records (452 units) in 2020 to 24,477 records (571 units) in 2024 (Table 1).

**Table 1:** Sample characteristics overall, by sex, and by survey year.

Characteristic	Overall	Male	Female	
Records, *n*	106,461	44,240	62,221	
Age, years	20.67 ± 1.54	20.79 ± 1.55	20.59 ± 1.53	
BMI, kg/m^2^	21.64 ± 3.52	22.39 ± 3.86	21.11 ± 3.17	
Total score	71.60 ± 8.56	68.80 ± 8.72	73.59 ± 7.86	
Survey year	Records, *n*	Units, *n*	Mean size	Total score
2020	17,302	452	38.3	70.72
2021	18,195	490	37.1	73.37
2022	23,003	536	42.9	69.86
2023	23,484	542	43.3	71.00
2024	24,477	571	42.9	73.11

Class-year units: *n* = 2,591; mean size 41.09 ± 12.63 (median 41, range 5–84); mean proportion male 0.406 ± 0.257; mean class BMI 21.63 ± 0.81 kg/m^2^.

**Figure 2:**
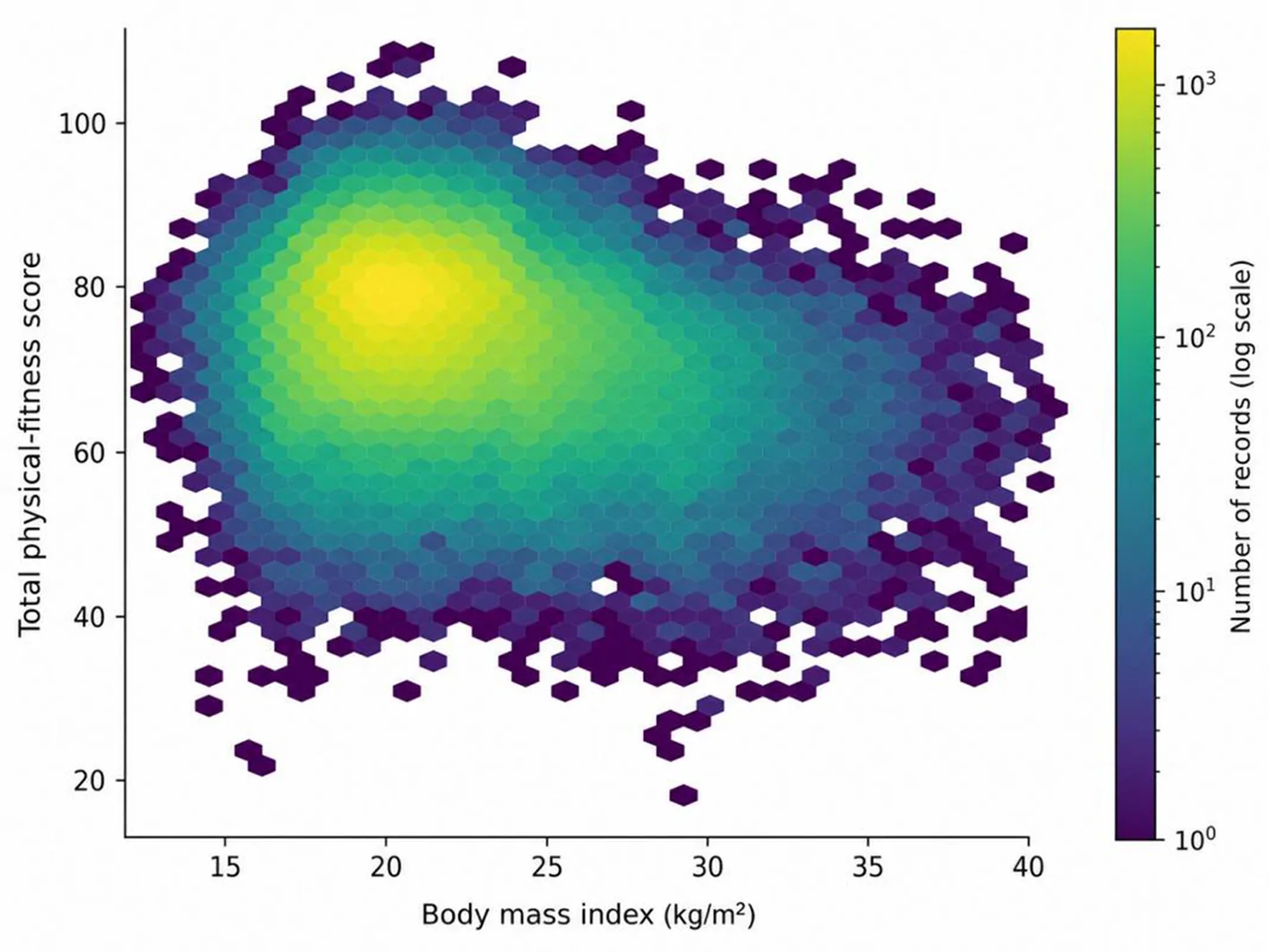
Hexbin density of individual BMI vs. total physical-fitness score across all 106,461 records, illustrating the joint distribution of BMI and total physical-fitness score.

The overall joint distribution of individual BMI and total physical-fitness score across the analytic sample is shown in Figure 2.

### 3.2 Included vs. excluded records

A total of 980 records were excluded during data cleaning. Compared with included records, excluded records were on average older (22.71 vs. 20.67 years, *p* < 0.001) and had higher BMI (22.93 vs. 21.64 kg/m^2^, *p* < 0.001) and a higher proportion of male students (68.1% vs. 41.6%, χ^2^ *p* < 0.001), reflecting that most exclusions were fifth-year or repeat students whose grade could not be validly assigned. Crucially, the mean total fitness score did not differ between included and excluded records (71.60 vs. 71.62; *t*-test *p* = 0.946), indicating that exclusions were unlikely to bias the outcome distribution (Table 2).

**Table 2:** Comparison of included and excluded records.

Variable	Included (*n* = 106,461)	Excluded (*n* = 980)	*p*
Age, years	20.67 ± 1.54	22.71 ± 1.57	< 0.001
BMI, kg/m^2^	21.64 ± 3.52	22.93 ± 6.88	< 0.001
Male, %	41.6	68.1	< 0.001
Total score	71.60 ± 8.56	71.62 ± 8.40	0.946

### 3.3 Between-class variation (null model)

The null random-intercept model yielded a between-class variance of 13.08 and a residual variance of 60.23, giving an ICC of 0.178. Thus roughly 18% of the total variance in individual fitness scores lay between class-year units, indicating substantial clustering and confirming that a multilevel approach was warranted. Between-class variation was present in every survey year when models were fitted separately (null-model ICC 0.097–0.253; Section 3.7 and Supplementary Table S1), showing that the clustering is not an artifact of pooling across years.

### 3.4 Fixed-effect associations (Model 2, primary specification)

In the primary specification (Model 2), higher within-class BMI was associated with lower fitness (β = −0.528, 95% CI −0.540 to −0.515, *p* < 0.001), as were older age (β = −0.128, *p* < 0.001) and male sex (β = −4.174, 95% CI −4.280 to −4.068, *p* < 0.001). Relative to first-year students, second-year (β = 0.803, *p* < 0.001) and fourth-year students (β = 1.913, *p* < 0.001) scored higher, whereas third-year students did not differ significantly (β = 0.283, *p* = 0.078). At the class level, a higher proportion of male students was associated with higher fitness (β = 1.090, 95% CI 0.557 to 1.622, *p* < 0.001), while larger class size (β = −0.012, *p* = 0.021) and higher mean class BMI (β = −0.906, 95% CI −1.080 to −0.731, *p* < 0.001) were associated with lower fitness. Calendar-year effects were non-monotonic, with 2024 (β = 3.057) and 2021 (β = 2.805) highest relative to 2020 and 2022 slightly lower (β = −0.372, *p* = 0.079), supporting categorical rather than linear modeling of year. Standardized coefficients indicated that sex (β^*^ = −0.240) and within-class BMI (β^*^ = −0.212) were the strongest individual-level correlates, followed by the 2024 and 2021 year contrasts (β^*^ = 0.150 and 0.123) and fourth-year grade (β^*^ = 0.092); mean class BMI was the largest class-level correlate (β^*^ = −0.084). The fixed effects accounted for 15.4% of the variance (marginal *R*^2^ = 0.154), and the full model including random intercepts accounted for 28.2% (conditional *R*^2^ = 0.282). Full estimates are given in Table 3.

**Table 3:** Model 2 (primary specification) fixed effects: unstandardized coefficients (B), 95% CIs, standardized coefficients (β^*^), and *p*-values.

Predictor	B	95% CI	β^*^	*p*
Intercept	71.291	70.922, 71.661	—	< 0.001
Grade 2 (vs. 1)	0.803	0.494, 1.111	0.042	< 0.001
Grade 3 (vs. 1)	0.283	−0.032, 0.598	0.014	0.078
Grade 4 (vs. 1)	1.913	1.577, 2.250	0.092	< 0.001
Sex (male vs. female)	−4.174	−4.280, −4.068	−0.240	< 0.001
BMI (within-class)	−0.528	−0.540, −0.515	−0.212	< 0.001
Age (centered)	−0.128	−0.172, −0.085	−0.023	< 0.001
Class size (centered)	−0.012	−0.023, −0.002	−0.017	0.021
Prop. male (centered)	1.090	0.557, 1.622	0.033	< 0.001
Mean class BMI (centered)	−0.906	−1.080, −0.731	−0.084	< 0.001
Year 2021 (vs. 2020)	2.805	2.379, 3.230	0.123	< 0.001
Year 2022 (vs. 2020)	−0.372	−0.787, 0.043	−0.018	0.079
Year 2023 (vs. 2020)	1.062	0.632, 1.492	0.051	< 0.001
Year 2024 (vs. 2020)	3.057	2.642, 3.472	0.150	< 0.001

Reference categories: female (sex), Year 1 (grade), 2020 (survey year). Marginal *R*^2^ = 0.154; conditional *R*^2^ = 0.282.

**Figure 3:**
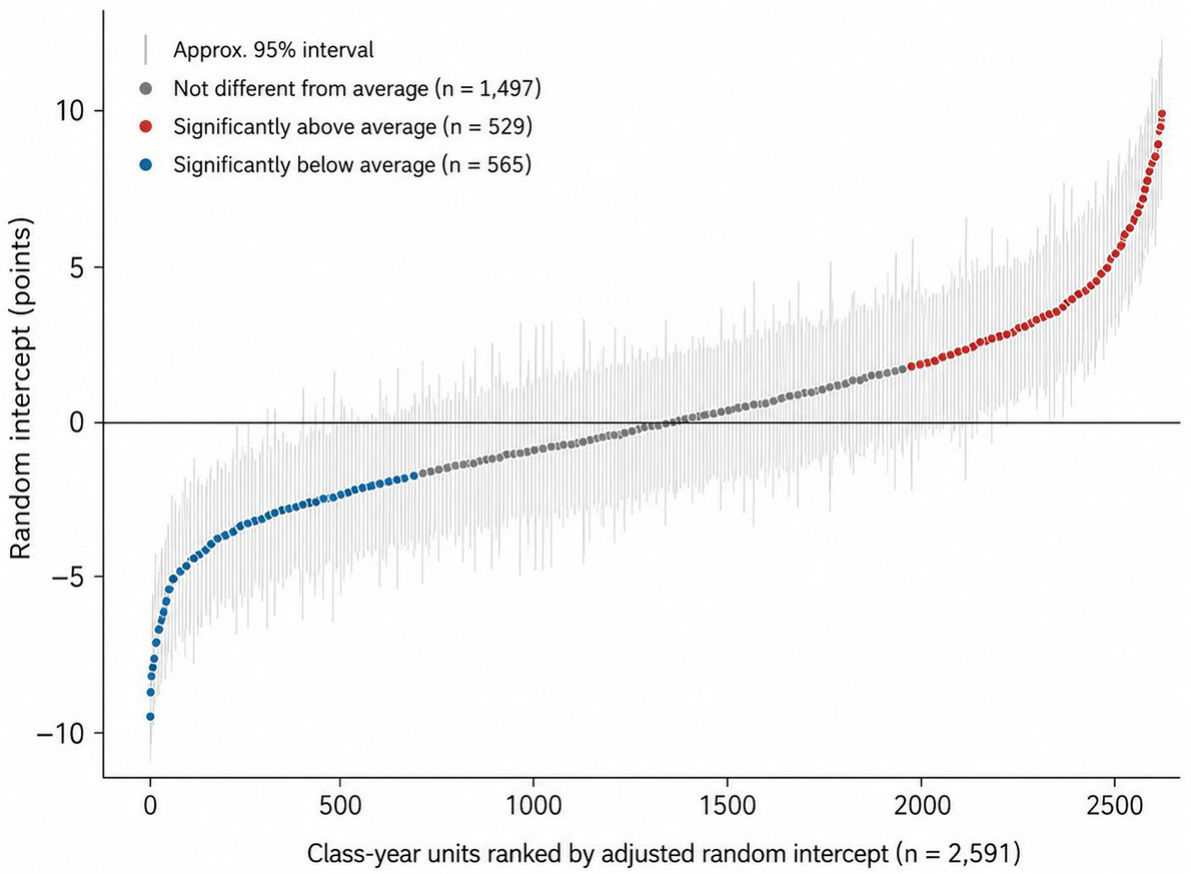
Caterpillar plot of class-year random intercepts (Model 2, empirical-Bayes estimates with 95% intervals). Units above the zero line score higher than average after adjustment (*n* = 529), units below score lower (*n* = 565), and units whose interval crosses zero are not distinguishable from the mean (*n* = 1,497). The intercepts span roughly 20 points.

Beyond the fixed effects, the adjusted class-year random intercepts from Model 2 spanned roughly 20 points (Figure 3), indicating substantial residual between-class variation that the measured individual and compositional predictors did not explain: 529 units scored significantly above and 565 significantly below the adjusted average, while the remaining 1,497 were not distinguishable from it.

### 3.5 Model comparison

Adding individual- and class-level covariates (Model 1) improved fit markedly over the null model (LR χ^2^ = 14,656.80, df = 9, *p* < 0.001). Adding calendar-year fixed effects (Model 2) produced a further significant improvement (LR χ^2^ = 435.04, df = 4, *p* < 0.001) and the lowest BIC among the intercept-only-slope models, and was adopted as the primary specification. Adding a random slope for within-class BMI (Model 3) again improved fit (LR χ^2^ = 463.07, df = 2, *p* < 0.001), indicating statistically detectable variation across classes in the BMI-fitness association. The adjusted ICC from Model 2 was 0.151, showing that individual and class composition explained part—but by no means all—of the between-class variation observed in the null model (Table 4).

**Table 4:** Model comparison.

Model	ICC	AIC	BIC	LR test vs. previous
Null (random intercept)	0.178	744,259	744,288	—
Model 1 (+ covariates)	0.177	729,621	729,735	χ^2^ = 14,656.80, df = 9^***^
Model 2 (+ year)	0.151	729,193	729,347	χ^2^ = 435.04, df = 4^***^
Model 3 (+ random slope)	—	728,734	728,907	χ^2^ = 463.07, df = 2^***^

All models fitted by maximum likelihood. ^***^p < 0.001.

### 3.6 Random-slope heterogeneity and covariance test

**Figure 4:**
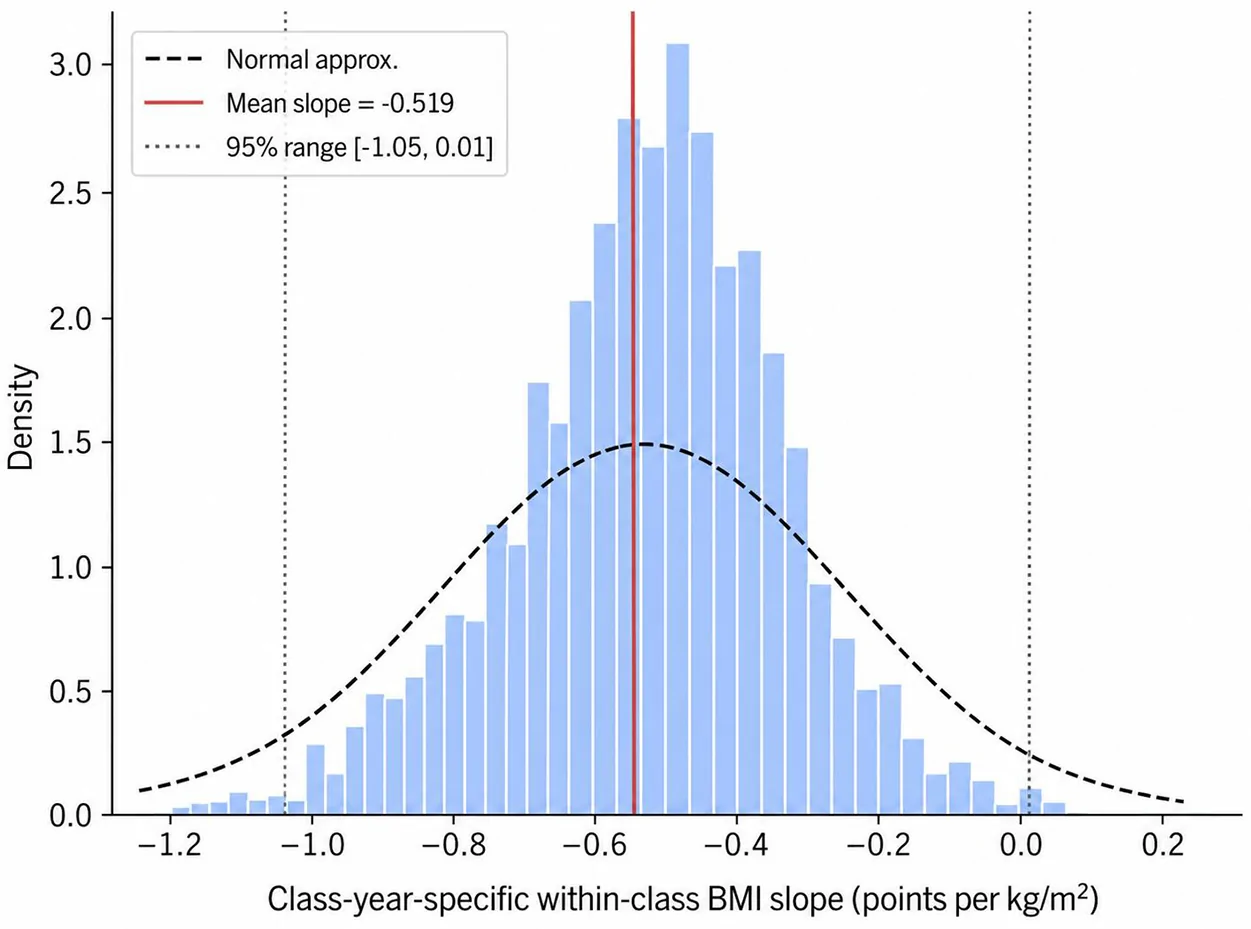
Distribution of class-specific within-class BMI slopes (Model 3, empirical-Bayes estimates). The mean slope is −0.52 and ~95% of slopes fall between −1.05 and +0.01; fewer than 4% are positive.

In Model 3 the within-class BMI slope varied across class-year units, with an estimated slope variance of 0.073 (SD ≈ 0.27). The mean slope was −0.519, and approximately 95% of class-specific slopes fell between −1.05 and +0.01; fewer than 4% of units had a positive empirical-Bayes slope, so the negative within-class BMI-fitness association held in almost all classes while varying in magnitude (Figure 4). Although this heterogeneity was statistically significant, its practical scale was modest. The covariance between the random intercept and the random BMI slope was small and not statistically significant (covariance = 0.039; correlation = 0.048; LR test of the full vs. a diagonal covariance structure χ^2^ = 1.46, df = 1, *p* = 0.228). Because this covariance component was not significant, the more parsimonious random-intercept Model 2 was retained for primary inference, with Model 3 reported as a robustness check.

### 3.7 Diagnostics and sensitivity analyses

Variance inflation factors for all Model 2 fixed effects were low (all < 2.1; maximum 2.03 for the 2023 contrast), indicating no problematic multicollinearity. Residual-vs.-fitted plots showed no marked heteroscedasticity, and the normal Q-Q plot indicated approximately normal residuals with mild left skew (−0.48) that is inconsequential at this sample size (Supplementary Figure S1). A cluster-level influence analysis identified 14 class-year units with standardized random intercepts beyond ±3 SD (empirical-Bayes intercepts ranged from −9.78 to +9.71 across all units); refitting Model 2 after removing these units left all key coefficients essentially unchanged (e.g., mean class BMI −0.906 → −0.934; sex −4.174 → −4.150; proportion male 1.090 → 1.224; within-class BMI −0.528 → −0.529).

Because individual students could not be linked across years, the model was refitted separately within each survey year (each student contributing at most one record). Substantial between-class variation was present in all five years (null-model ICC 0.097–0.253), and the direction of the key associations was stable: within-class BMI, mean class BMI, and male sex were negative and significant in every year, while the proportion of male students was positive and significant in 2022 and 2023 and non-significant in 2020, 2021, and 2024 (Table 5; full estimates in Supplementary Table S1). The magnitude of the sex coefficient varied across years (from −0.45 in 2020 to −9.00 in 2022), mirroring a genuine year-to-year fluctuation in the raw male-female score gap; this non-monotonic pattern reinforces the decision to model calendar year categorically. Additional models that added mean class age as a level-2 predictor, or that removed age or grade, left the class-level associations robust (e.g., with mean class age added, proportion male = 0.966 and mean class BMI = −0.984; auxiliary age-grade *R*^2^ = 0.269, VIF = 1.37), indicating that age and grade did not induce material collinearity.

**Table 5:** Single-year sensitivity analysis: between-class ICC and key coefficients by survey year (each student contributes at most one record).

Year	ICC null	ICC adj	BMI (w/in)	Sex	Prop male	Mean BMI
2020	0.097	0.093	−0.455^***^	−0.448^***^	−0.890	−0.451^*^
2021	0.132	0.084	−0.526^***^	−4.771^***^	0.096	−0.553^***^
2022	0.253	0.168	−0.538^***^	−9.003^***^	2.718^***^	−1.718^***^
2023	0.129	0.092	−0.644^***^	−4.332^***^	2.027^***^	−1.726^***^
2024	0.117	0.080	−0.461^***^	−1.348^***^	0.732	−0.291^*^

^*^p < 0.05; ^***^ *p* < 0.001. “Prop male” and “Mean BMI” are class-level (level-2) coefficients; “BMI (w/in)” is the within-class individual coefficient.

## 4 Discussion

### 4.1 Summary of main findings

Using 106,461 annual surveillance records from 2,591 class-year units at one Chinese university, this study documented three main findings. First, between-class variation in physical fitness was substantial: about 18% of the variance in total scores lay between class-year units, and meaningful between-class variation was present in every survey year. Second, after adjustment for individual characteristics and calendar year, both individual and class-compositional factors were associated with fitness—within-class BMI, age, and male sex negatively at the individual level; and, at the class level, mean class BMI and class size negatively and the proportion of male students positively. Third, the within-class BMI-fitness association varied across classes, though the variation was modest in magnitude and almost always negative in direction. These findings are descriptive and compositional; they identify where variation lies rather than why it arises.

### 4.2 Between-class variation and its relevance to health psychology and research design

The ICC of 0.178 indicates that the class-year unit is a non-trivial level of clustering in university-student fitness. This is consistent with Novak et al. (2026), who reported substantial school-level variation in youth fitness that persisted after adjustment for sex and class, and with evidence that proximal educational settings shape students' activity and health behavior (Zhou et al., 2025; Cui et al., 2024). From a health-psychology perspective, the class is a plausible carrier of peer norms, motivational climate, and collective efficacy (Bandura, 1997; Ryan and Deci, 2000; Zaccaro et al., 1995): students in the same class share schedules, physical-education instructors, facilities, and social comparisons, any of which could generate correlated fitness outcomes. We stress, however, that our data contain no direct measures of these constructs; the between-class variation we document is an empirical target that such mechanisms could later be tested against, not evidence for them. Methodologically, the sizeable ICC also carries a clear implication: single-level analyses of university-student fitness that ignore class clustering will understate standard errors and risk over-stating the precision of individual-level associations. Even where class context is not the substantive interest, modeling it is necessary for valid inference.

### 4.3 Individual-level correlates

The negative within-class BMI coefficient reproduces, at the individual level, the well-documented inverse BMI-fitness association in Chinese students (Chen et al., 2020; Wu et al., 2024; Zhang et al., 2018). Because BMI also contributes 15% of the NSPFS total score directly, part of this association is definitional; the remainder reflects the poorer performance of higher-BMI students on endurance, speed, and strength components. Standardized coefficients placed sex and within-class BMI as the two strongest individual-level correlates. The sex coefficient must be read in light of the sex-specific scoring norms: because norms already adjust for sex, the residual male disadvantage in total score reflects the specific weighting of components rather than a simple fitness gap, and its magnitude varied across years with the raw score gap. The higher scores of second- and fourth-year relative to first-year students may reflect survivor and adaptation effects, whereby students become familiar with testing and those with very low fitness are progressively less represented; the absence of a monotonic grade gradient cautions against a simple maturation interpretation.

### 4.4 Compositional vs. contextual class-level associations

At the class level, higher mean BMI was the strongest compositional correlate of lower fitness, and a higher proportion of male students was associated with higher average scores, while larger classes scored marginally lower. It is essential to interpret these as compositional rather than contextual associations. Mean class BMI is an aggregate of the individual BMIs within the unit, and the proportion of male students summarizes its sex composition; both may capture who is in the class rather than any causal property of the class environment. The class-size association, though statistically significant, was practically negligible—about a 0.24-point difference in total score across a 20-student difference in class size—and should not be over-interpreted. Separating within-class from between-class BMI associations through centring (Enders and Tofighi, 2007) clarified that individual deviations from the class mean and the class mean itself were each independently associated with fitness, but it cannot by itself distinguish composition from context. Distinguishing genuine contextual effects (e.g., a shared motivational climate) from compositional ones would require class-level measures of the hypothesized mechanisms, which were not available here.

### 4.5 Practical implications

Two cautious implications follow. For research and surveillance, fitness data from universities should be analyzed with multilevel or cluster-robust methods, and reports should present between-class variation rather than only pooled individual associations. For practice, the concentration of lower fitness within particular class-year units—some scoring nearly 10 points below average—suggests that class-level screening could help target physical-education resources, provided that any targeting is based on directly measured needs rather than on sex or BMI composition, to avoid stigmatizing groups. Because the class-level associations here are compositional, they justify identifying which classes differ, not attributing those differences to the class environment.

### 4.6 Strengths and limitations

This study has notable strengths: a very large multi-year surveillance sample, a full reproduction of the analytic pipeline from raw records, explicit within/between separation of BMI, and extensive diagnostic and sensitivity analyses. Several limitations must be acknowledged. First and most important, the design is cross-sectional at the record level: because a stable cross-year identifier was not available, students could not be linked longitudinally, within-student correlation across years could not be modeled, and no causal or developmental inference is warranted; the annual single-year analyses partly mitigate but do not resolve this. Second, the class-level variables are compositional summaries, not measured features of the class environment, so the between-class variation we report should not be read as evidence of contextual or psychosocial effects. Third, no psychosocial, behavioral, lifestyle, or physical-activity variables (e.g., sleep, diet, training, motivation, peer norms) were available, so the fixed effects explain a limited share of variance (marginal *R*^2^ = 0.154) and residual confounding is likely. Fourth, the total score is a weighted composite that already embeds BMI and sex-specific norms, which shapes the BMI and sex coefficients. Fifth, the data come from a single university in one province, and test administration may have varied across the 2020–2024 window—including pandemic disruption—so generalisability is limited and calendar-year effects conflate secular, administrative, and measurement changes. Modeling year categorically limits, but does not remove, this concern.

### 4.7 Future directions

Future work should, first, obtain ethically governed stable identifiers to build genuinely longitudinal multilevel models of within-student fitness trajectories; second, measure class-level psychosocial and behavioral constructs directly—motivational climate, peer norms, collective efficacy, instructor characteristics, and facility access—to test whether they account for the between-class variation documented here; third, extend the analysis to multiple institutions and provinces to assess generalisability and to add higher levels (major, college, institution) to the hierarchy; and fourth, complement the composite total score with component-specific outcomes to separate definitional from behavioral pathways. Such studies would move the field from documenting that classes differ to explaining why.

## 5 Conclusion

In a large multi-year surveillance sample from one Chinese university, physical-fitness scores varied substantially between class-year units (ICC = 0.178), and both individual characteristics (BMI, age, sex, grade) and class composition (mean BMI, proportion male, class size) were independently associated with fitness. The within-class BMI-fitness association was negative in almost all classes but varied modestly in strength. These results establish class-level clustering as a robust, reproducible feature of university-student fitness data and generate testable hypotheses—that peer norms, motivational climate, and collective efficacy contribute to between-class differences—for future studies that measure such mechanisms directly. Analytically, they show that valid inference about university-student fitness requires methods that account for the clustered structure of the data.

## Data Availability

The de-identified data supporting the findings of this study are available from the corresponding author upon reasonable request, subject to institutional approval and applicable data-protection requirements.
